# Rapid and quantitative antimalarial drug efficacy testing via the magneto-optical detection of hemozoin

**DOI:** 10.1038/s41598-020-70860-y

**Published:** 2020-08-20

**Authors:** Petra Molnár, Ágnes Orbán, Richard Izrael, Réka Babai, Lívia Marton, Ádám Butykai, Stephan Karl, Beáta G. Vértessy, István Kézsmárki

**Affiliations:** 1 Malaria Research Laboratory, Institute of Enzymology, Research Centre for Natural Sciences, Budapest, 1117 Hungary; 2 Department of Physics, BME Budapest University of Technology and Economics, Budapest, 1111 Hungary; 3 Doctoral School of Multidisciplinary Medical Sciences, University of Szeged, Szeged, 6720 Hungary; 4 Department of Applied Biotechnology and Food Sciences, BME Budapest University of Technology and Economics, Budapest, 1111 Hungary; 5 Australian Institute of Tropical Health and Medicine, James Cook University, 1/14-88 McGregor Road, Smithfield, QLD 4870 Australia; 6 Vector-Borne Diseases Unit, PNG Institute of Medical Research, P.O. Box 378, Madang, 511 Madang Province Papua New Guinea; 7 Experimental Physics 5, Center for Electronic Correlations and Magnetism, Institute of Physics, University of Augsburg, 86159 Augsburg, Germany

**Keywords:** Diagnostic markers, Malaria, Biological physics, Diagnostics, Parasite biology

## Abstract

Emergence of resistant *Plasmodium* species makes drug efficacy testing a crucial part of malaria control. Here we describe a novel assay for sensitive, fast and simple drug screening via the magneto-optical detection of hemozoin, a natural biomarker formed during the hemoglobin metabolism of *Plasmodium* species. By quantifying hemozoin production over the intraerythrocytic cycle, we reveal that hemozoin formation is already initiated by ~ 6–12 h old ring-stage parasites. We demonstrate that the new assay is capable of drug efficacy testing with incubation times as short as 6–10 h, using synchronized *P. falciparum* 3D7 cultures incubated with chloroquine, piperaquine and dihydroartemisinin. The determined 50% inhibitory concentrations agree well with values established by standard assays requiring significantly longer testing time. Accordingly, we conclude that magneto-optical hemozoin detection provides a practical approach for the quick assessment of drug effect with short incubation times, which may also facilitate stage-specific assessment of drug inhibitory effects.

## Introduction

Drug resistance constitutes a long-standing major issue in the fight against malaria. Adaptation of *Plasmodium* parasites to antimalarial drugs poses a serious threat to both existing drugs and new drug candidates. As a recent example, the development of artemisinin-based drugs was honored by the Nobel Prize in Medicine 2015, yet, resistance of *Plasmodium* against artemisinins and artemisinin combination therapies may soon become a global issue, as documented by annual reports of WHO^1^. Thus, continuous surveillance of parasite resistance is of utmost concern, for which treatment-efficacy studies, the analysis of molecular markers and in vitro*/*ex vivo drug susceptibility assays are used, providing the scientific background for treatment guidelines^2–4^. Since it excludes a range of host-specific effects influencing treatment efficacy, in vitro testing is an essential tool to study drug resistance, facilitating high reproducibility due to well-controlled measurement conditions. Various methods have been developed in order to make in vitro drug susceptibility testing faster, lower-cost and more convenient^5^. The targets of antimalarial drug treatment are the asexual intraerythrocytic life-cycle stages of the parasites, and in vitro drug susceptibility assays quantify the maturation and multiplication of these stages under drug effect. After a merozoite invades a red blood cell (RBC), the parasite undergoes maturation through the ring, trophozoite and schizont stages, which altogether takes approximately 48 h. This is followed by the invasion of new RBCs, which completes the intraerythrocytic life cycle^6^.

**Figure 1:**
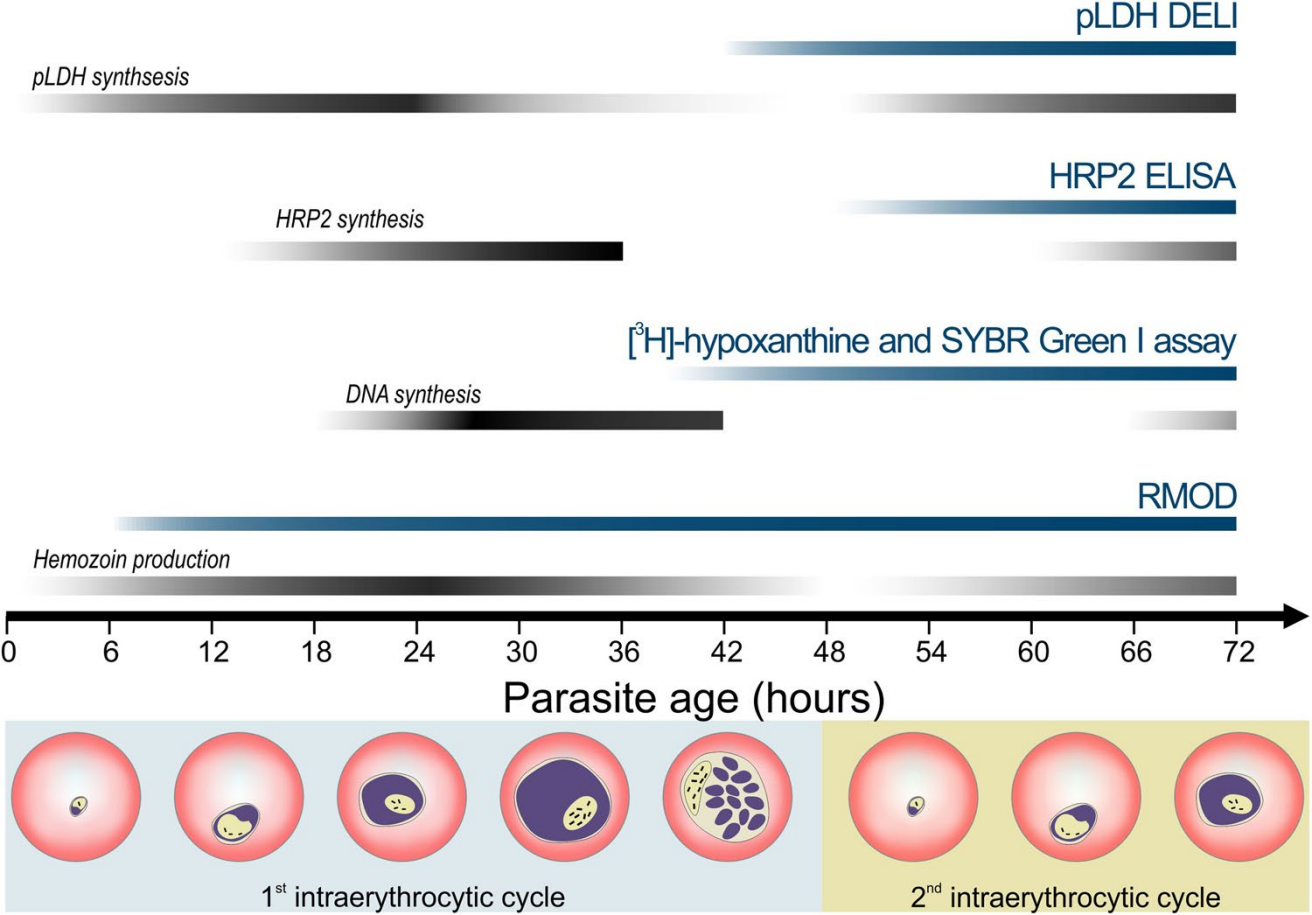
Comparison of drug susceptibility assay methods and their principle of detection. Blue and black stripes indicate the time intervals when the corresponding assays are typically carried out and when the detected parasite products are formed, respectively. Pictures below the chart represent parasite maturation during the 48 h of the intraerythrocytic cycle. The *P. falciparum* HRP2 and pLDH proteins are detected by ELISA and DELI methods. The HRP2 is secreted mostly during the second half of the intraerythrocytic cycle, while the pLDH expression starts during the early stages and finishes by the schizont stage^9,10,60,61^. The fluorescent SYBR Green dye is added to the samples after the incubation time to intercalate with the parasitic DNA^33^. The RMOD assay exploits the detection of hemozoin, produced by the hemoglobin metabolism of the parasites from early hours of the intraerythrocytic cycle^15,26,43^ until the schizont stages.

Current in vitro drug susceptibility assays mostly rely on the quantification of different protein markers, DNA, or metabolic products, which are indicators of the overall parasite biomass. Examples for such assays include the microscopic evaluation of parasite maturation, use of radioactive markers or intercalating fluorescent dyes, and detection of parasite-specific proteins^2,7,8^. Some of the most frequently used techniques include the HRP2 ELISA assay, the SYBR Green I assay, the [^3^H]-hypoxanthine incorporation assay and the pLDH assay which are displayed together with their target of detection in Fig. 1. The HRP2 and pLDH assays measure the *P. falciparum* histidine-rich protein 2 or parasite lactate dehydrogenase proteins utilizing colorimetric ELISA-based approaches. The application of HRP2 assay for field isolates may be increasingly hindered by the spread of HRP2 deletion, and both methods rely on relatively costly monoclonal antibodies^5,8–10^. Many assays rely on a DNA intercalating dye, which is a sensitive marker of parasite maturation. The radioactive [^3^H]-hypoxanthine assay measures the incorporation of the aforementioned material into parasitic DNA during replication, which occurs between the trophozoite and early schizont stages. While the method has a high sensitivity, the use of radioactive materials requires specific careful handling, making it a less appealing alternative for many users^11^.

Despite a variety of available techniques to quantify parasite biomass, the applied incubation times are usually in the range of 48–72 h, though there are clear efforts to shorten the incubation time and to develop assays aiming for early intraerythrocytic stages^12,13^. Furthermore, a method capable of detecting inhibitory action rapidly and in a stage-specific fashion might be advantageous in ex vivo field assays as well. The fast assessment of parasite resistance in case of patient isolates could support individual treatment strategies. The optimized drug choice can facilitate the faster recovery of patients and also could contribute to drug resistance management.

The rotating-crystal magneto-optical diagnostic (RMOD) technique has been previously reported to be an efficient and highly sensitive tool that detects and quantifies hemozoin produced by the intraerythrocytic stages of malaria parasites^14–17^. Here, we describe the application of the RMOD method for the quantification of hemozoin produced by the laboratory-adapted *P. falciparum* 3D7 strain within a single life cycle. Building on this, we demonstrate that the RMOD method can also be used as a cost-efficient, practical and sensitive tool for the in vitro assessment of drug susceptibility with considerably shorter incubation times than those typically employed in assays of similar or higher complexity. Since our assay is based on the detection of a natural biomarker, which is accumulated throughout subsequent intraerythrocytic stages and subsequent intraerythrocytic cycles of in vitro parasite cultures, it is capable to monitor drug efficacy from early to late stages of parasite development within a single cycle as well as slow drug effects through multiple cycles.

## Results

### Tracking parasite maturation via the hemozoin production rate

**Figure 2:**
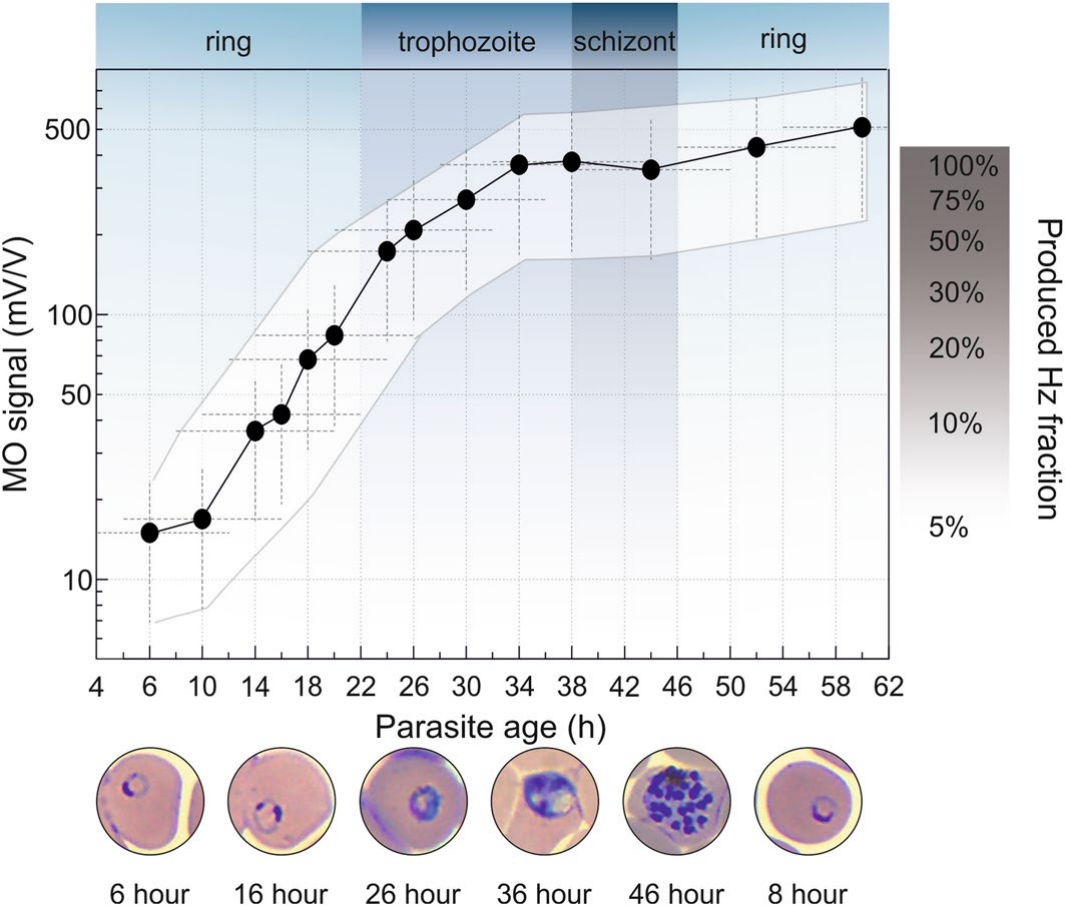
Typical hemozoin production characteristics of *P. falciparum 3D7*. The black circles represent the average MO values measured on drug-free parasite cultures of 1% parasitemia as a function of the mean parasite age (for details see “Sample preparation for RMOD measurements” section.). The white area shows the typical error band of the hemozoin production curve. This includes the horizontal error due to the finite age distribution of the cultures and the uncertainty of age assessment, as well as the vertical error due to the scatter of MO values originating from independent assays (for details see “Optical microscopy and smear evaluation” section.). The blue/grey background shading indicates the development of the subsequent erythrocytic stages^55,62^. On the right, the progress of hemozoin formation is shown in percentages of the full amount produced during the first erythrocytic cycle. The microscopy images of infected RBCs shown below the graph are representative of the developmental stages observed at the corresponding time points.

We analyzed the hemozoin production characteristics of the *P. falciparum* 3D7 strain by quantifying the overall amount of hemozoin produced by synchronized cultures of 1% parasitemia, as shown in Fig. 2.

The MO signal measured at the first time point includes three contributions: Hemozoin crystals already produced by the hemoglobin metabolism of early rings, hemozoin produced by the few older parasites inherently present due to imperfect synchronization, and a small fraction of the hemozoin that was produced in previous cycles and could not be completely removed by our sample preparation methodology. Based on the synchronization window of 8 ± 4 h, characteristic to the cultures at the beginning of the assay, and the numerous washing steps applied before assay initiation, we presume that most of the hemozoin detected at assay initiation was produced by the parasites of the actual intraerythrocytic cycle. A steep increase in hemozoin concentration is observable starting from ~ 6–12 h of parasite age and lasting until ~ 34 h. The corresponding parasite stages, i.e., the mid-late ring and the first half of the trophozoite stages can be classified as the G1 phase of the cell cycle of *P. falciparum*, suggesting an increased rate of metabolic processes taking place during this time^18–20^. After ~ 34 h, the late trophozoite stage is the period of DNA replication, aka. the S phase. It is followed by the schizont stage, when the packaging of genetic material occurs to create new merozoites and, thus, low hemoglobin metabolism is expected^18–20^. In fact, our analysis shows that 25 ± 15% and 65 ± 15% of the total hemozoin is produced during the ring and the trophozoite stage, respectively, while less than 10% of hemozoin production is observed during the schizont stage. In summary, the hemozoin production characteristics determined by the RMOD method are in accordance with the primary functions of the different developmental stages and the previously proposed hemozoin production characteristics^21–26^. After the schizonts rupture, the hemozoin is released and the merozoites start a new cycle by invading new red blood cells. Following the invasion, hemozoin production of the young rings becomes visible in the MO signal after ~ 6–10 h of parasite age. From this point, the previously described characteristics drive the hemozoin production of the synchronized parasite population.

### Quantifying antimalarial effect at short incubation times

In order to determine the shortest incubation time necessary for the accurate assessment of IC50 values, we performed drug susceptibility assays with well-characterized antimalarial drugs, namely chloroquine, piperaquine and dihydroartemisinin, on tightly synchronized cultures.

**Figure 3:**
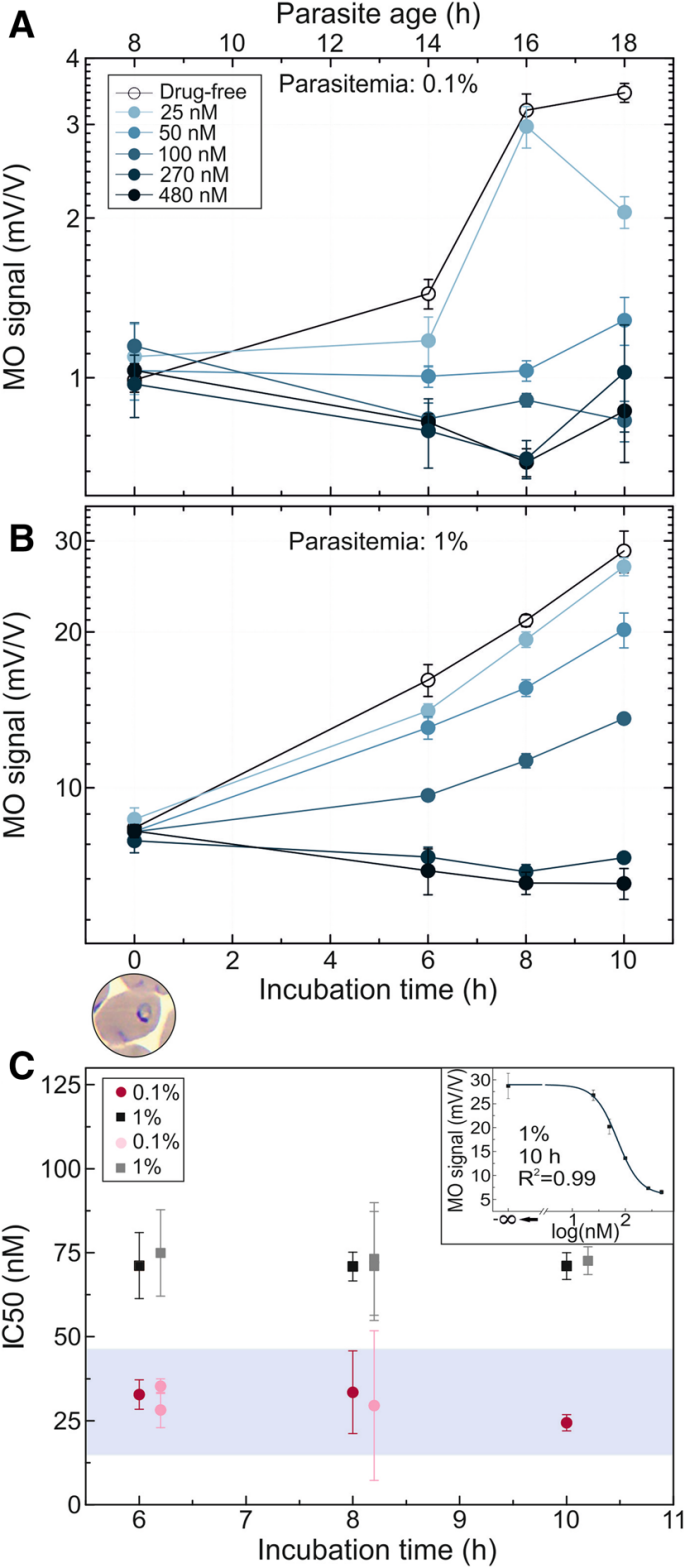
Inhibitory effect of piperaquine on *P. falciparum* 3D7 cultures as detected by RMOD assay. **A**,**B** MO values as a function of incubation time for the cultures with 0.1% and 1% parasitemia, respectively, incubated with various drug concentrations. The top axis shows the age of the parasites at the given sampling points determined by optical microscopic evaluation. Below the graph, an image shows the typical parasite stage at the starting point of the assay. The color coding of the curves indicates increasing drug concentrations from light to dark shades. The empty circles represent the drug-free control samples. Each data point represents the average of the MO values measured on triplicates. **C** IC50 values determined after 6, 8 and 10 h of incubation. Black and grey squares represent the IC50 values determined for the assay at 1% parasitemia in **B** and another independent assay of similar conditions, respectively. Red and pink circles show the IC50 values obtained from the assay of 0.1% parasitemia in **A** and another independent assay of similar conditions, respectively. The blue shaded area indicates the range of IC50 values reported in literature (Supplementary Table S1.)^29–33^. A representative dose–response fit curve corresponding to the 10 h time point of 1% assay is displayed in the top right corner of **C**.

Five concentrations of piperaquine were tested using a *P. falciparum* 3D7 cell culture set to 1% parasitemia, with the majority of the parasites being approximately 8-h old rings at the beginning of incubation (Fig. 3a). Another assay was carried out using the same culture diluted to a starting parasitemia of 0.1% (Fig. 3b). Therefore, the stage distributions of the two cultures can be regarded identical in the sampling time points.

The first sampling was carried out after 6 h of incubation. The drug effect was clearly detected even after this short incubation, as reflected by the significant difference (*p* < 0.001) between the MO signal of the drug-free control and the samples with mid-range drug concentrations. The piperaquine treatments of 270 nM and 480 nM concentrations had a complete inhibitory effect, i.e., no increase of the MO signal was observed with respect to its initial value and weak inhibition was even observed for the lowest (25 nM) concentration. The same tendency was followed at samplings after 8 and 10 h.

The IC50 values for the piperaquine assays with 1% parasitemia determined by the RMOD method after 6, 8 and 10 h of incubation show small variance, as seen in Fig. 3c, with an average (IC50 = 72.9 ± 1.59 nM) that is somewhat higher than the typical IC50 values of piperaquine reported earlier (Fig. 3c and Supplementary Table S1.)^27–33^. The average IC50 value determined from the assays at 0.1% parasitemia (IC50 = 30.61 ± 4.01 nM) is significantly lower compared to that obtained for 1% parasitemia (Fig. 3c). The phenomenon that the IC50 values of certain antimalarials show parasitemia dependence has previously been documented in in vitro and ex vivo assays by other research groups^34^.

**Figure 4:**
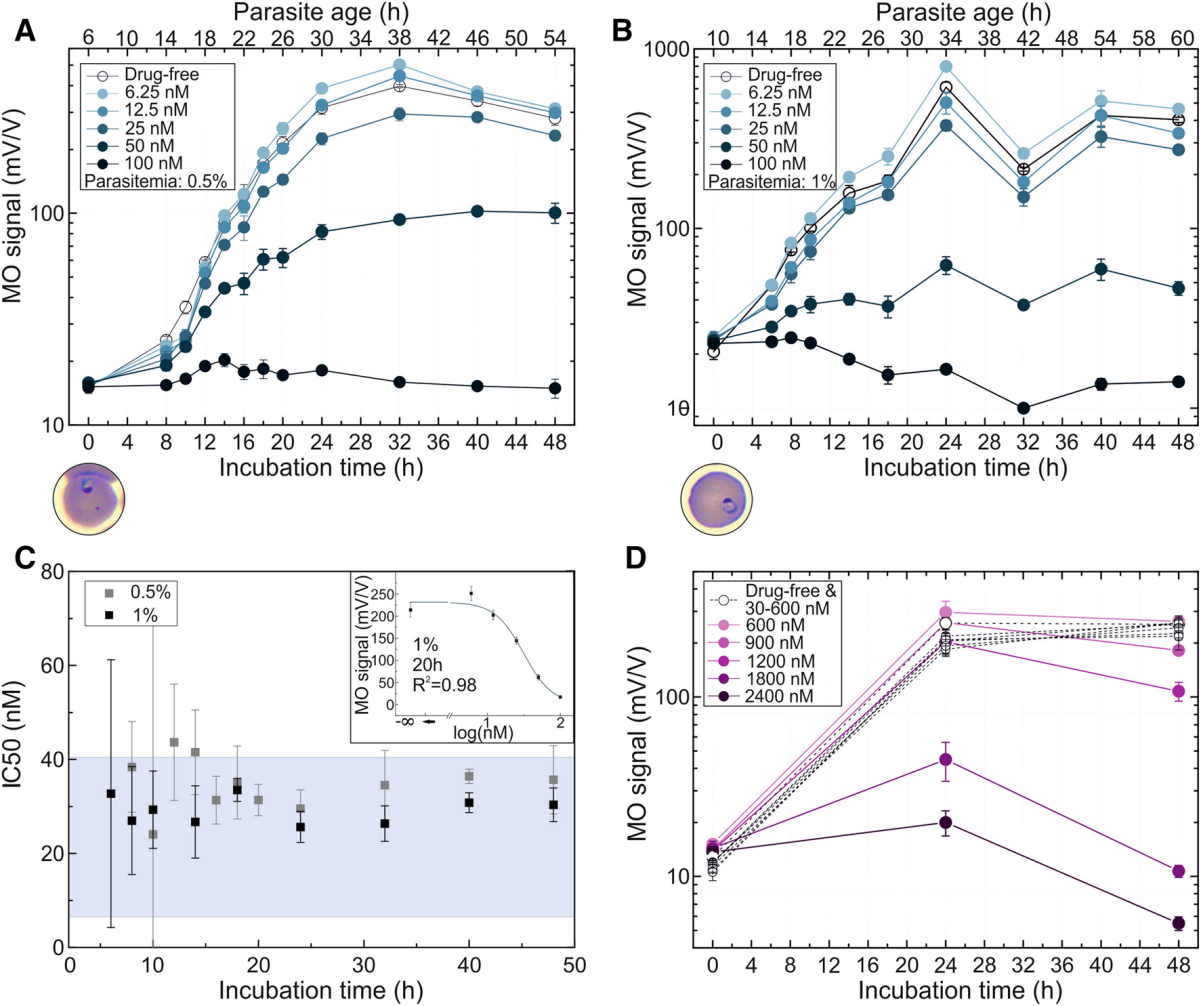
Inhibitory effect of chloroquine on *P. falciparum* 3D7 and W2 cultures as measured by RMOD assay. **A**,**B** MO values of the *P. falciparum* 3D7 culture with 0.5% and 1% parasitemia incubated with various drug concentrations as a function of incubation time up to 48 h. The top axis shows the age of the parasites at the given sampling points, determined by optical microscopic evaluation. Below the graph, an image shows the typical parasite stage at the starting point of the assay. The color coding of the curves indicates the increasing drug concentrations from light to dark shades. The empty circles represent the drug-free control samples. Each circle represents the average of the MO values measured on triplicates. **C** IC50 values as a function of incubation time. IC50 values and the standard errors of their fit are represented by black squares in case of the 1% assay and grey squares in case of the 0.5% assay. The blue shaded area indicates the range of formerly reported IC50 values (Supplementary Table S1.)^10,29–33,35,52–54^. The upper right corner shows a representative dose–response fit curve corresponding to the 20 h time point of the 1% assay. **D** MO values of the resistant *P. falciparum* W2 culture with 1% parasitemia incubated with various drug concentrations as a function of incubation time up to 48 h. The color coding of the curves indicates the increasing drug concentrations from light to dark shades. The empty squares represent the growth of samples treated with an ineffective drug dose (30–600 nM). Each circle represents the average of triplicates.

For the first chloroquine assay a *P. falciparum* 3D7 cell culture was set to 0.5% parasitemia of synchronized parasites, and the assay started when the majority of the parasites were approximately 6-h old rings (Fig. 4a). The most intense hemozoin production is observable between the 6 h and 24 h sampling points of the assay, corresponding to approx. 12-h old rings and 32-h old trophozoites, as determined by optical microscopy. For later time points a less intense hemozoin production is found, in accord with Fig. 2. At the last sampling point, besides mature schizonts, very young (0–4 h) rings were already present in the blood smears of the drug-free control, i.e., a certain portion of the population has started its second erythrocytic cycle. The drug action was again clearly detected at the first sampling point, i.e., after 8 h of incubation. The concentration of 100 nM had a complete inhibitory effect throughout the whole cycle. For intermediate concentrations, the drug effect weakened with decreasing concentration. The lowest (6.25 nM and 12.5 nM) concentrations did not suppress the growth of the parasites. Starting from 18 and 24 h of incubation, respectively, samples in these two assays even showed slightly higher MO values than the drug-free samples.

We have performed another assay using chloroquine, shown in Fig. 4b, where the starting parasitemia was set to 1%, and the majority of parasites were ~ 10-h old rings. Already after 6 h of incubation, the MO signals of culture samples treated with different drug concentrations are clearly distinguishable and a significant difference (*p* < 0.01) is found between the drug-free control and the samples with mid-range concentrations. The concentration of 100 nM had a complete inhibitory effect, while the lowest (6 nM) concentration did not influence the growth of the culture at this early sampling point and even slightly promoted the hemozoin production after longer incubation.

The IC50 values for chloroquine were calculated for each time point of assays at 0.5% and 1% parasitemia (Fig. 4c). The IC50 values determined after different incubation times show no characteristic time dependence; and the averages of the values measured in all time points are equal within their margin of error for the two assays (average IC50 for chloroquine assay with 0.5% parasitemia = 29.15 ± 2.71 nM and average IC50 for chloroquine assay with 1% parasitemia = 34.68 ± 5.28 nM). These values fall into the range reported in the literature (Fig. 4d and Supplementary Table S1.), typically determined in assays with incubation times of 48–72 h^31,33,35^.

In order to investigate chloroquine resistance by the RMOD assay, we performed measurements with the *P. falciparum* W2 chloroquine resistant strain at a parasitemia level of 1%. First, the lower limit of the concentration range was set to the average IC50 value of the sensitive *P. falciparum* 3D7 strain based on our previous measurements. As expected, there was no detectable difference in the hemozoin production of the drug-free and drug-treated culture aliquots after 24 h and 48 h of incubation (Fig. 4d). By further increasing the drug concentration, we have obtained an average IC50 value of 1,207.5 ± 240.5 nM, which is about 40-times higher than that of the sensitive *P. falciparum* 3D7 strain.

We have also assessed the performance of the RMOD assay for drug susceptibility screening with dihydroartemisinin, a compound with a different mechanism of action than the previously presented aminoquinolines^36–39^.

**Figure 5:**
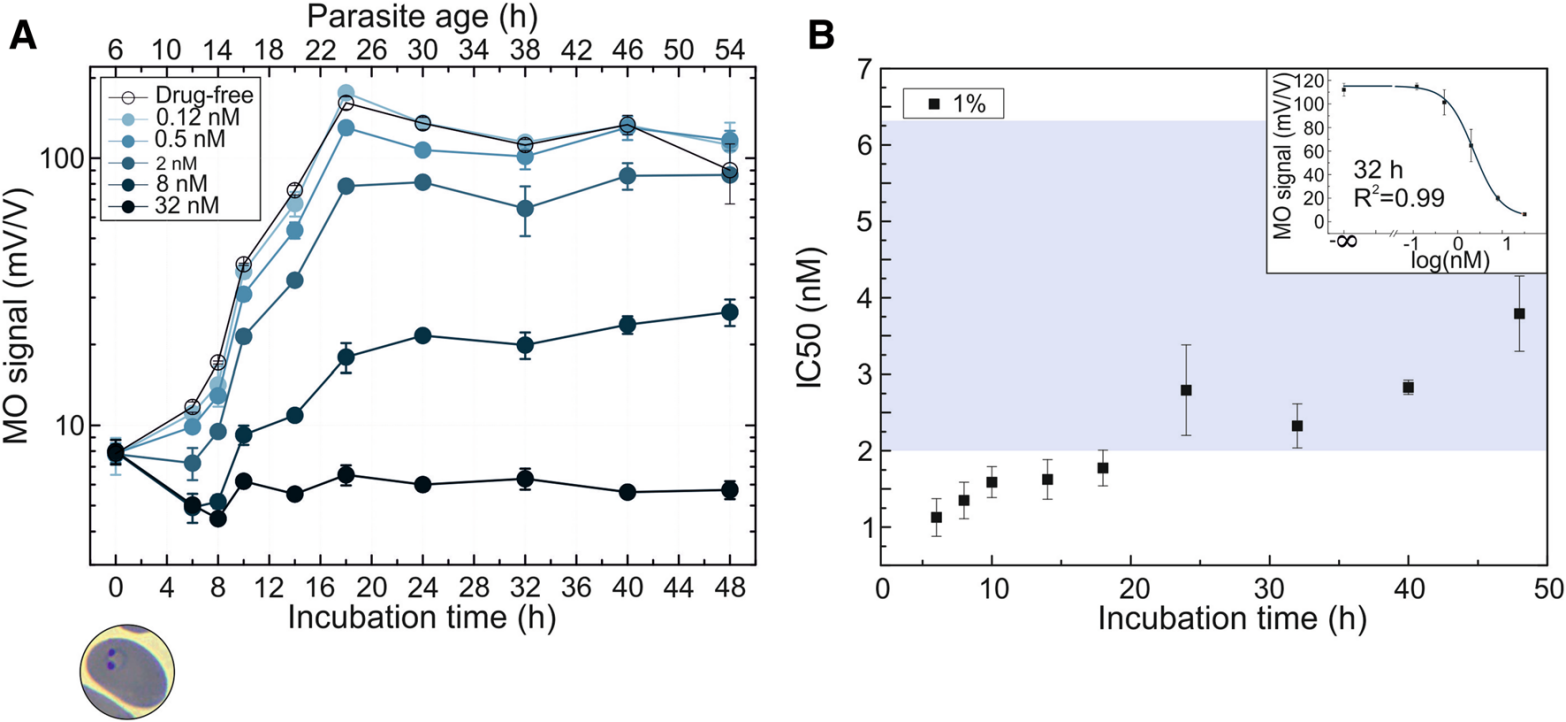
Inhibitory effect of dihydroartemisinin on *P. falciparum* 3D7 cultures as detected by RMOD assay. **A** MO values as a function of incubation time at 1% parasitemia incubated with various concentrations of dihydroartemisinin. The top axis shows the age of the parasites at the given sampling points, determined by optical microscopic evaluation. Below the graph, an image shows the typical parasite stage at the starting point of the assay. The color coding of the curves indicates the increasing drug concentrations from light to dark shades. The empty circles represent the growth of drug-free control samples. Each circle represents the average of the MO values measured on triplicates. **B** IC50 values of the dihydroartemisinin assay as a function of incubation time. The calculated values show a systematic increase during the complete intraerythrocytic cycle. The blue shaded area indicates the range of IC50 values reported earlier (Supplementary Table S1)^30–33^. The upper right corner shows a representative dose–response fit curve corresponding to the 32 h time point of the 1% assay.

In case of dihydroartemisinin, the culture was set to 1% parasitemia with ~ 6-h old rings at the beginning of the assay (Fig. 5.). For the drug-free samples, an intense hemozoin production is found between the 6 h and the 18 h time points, corresponding to 12-h old rings and 24-h old trophozoite stages. It is followed by a stagnation of the hemozoin level, where the parasites in this culture reached the early schizont stage at the 32 h time point and the beginning of the second erythrocytic cycle already started between the 40- and 48-h sampling points for a great proportion of the population.

Concerning the drug effect, the highest concentration showed complete inhibitory action, while the lowest concentration had no inhibitory effect. The hemozoin production of the cultures exposed to the in-between drug concentrations indicate a partially inhibited development and follow overall the expected course. The IC50 values, calculated for each time point, coincide well with those reported in the literature (Supplementary Table S1.)^30–33^.

## Discussion

Drug resistance presents a serious challenge to malaria control and elimination^7^. The RMOD assay offers a new, label-free approach to quantify the maturation of parasite populations. While the present work primarily aims at reducing the incubation time required for drug inhibition studies, the characteristics of intraerythrocytic hemozoin production by *Plasmodium* species make both short-term and long-term analysis feasible. Subsequent parasite stages of a single intraerythrocytic cycle are characterized by a steadily increasing hemozoin content. In case of in vitro cultures, hemozoin is further accumulated during subsequent cycles. Correspondingly, the RMOD method can follow parasite maturation within a single intraerythrocytic cycle with a sub-cycle resolution and can also be used to monitor parasite maturation over several cycles.

The high specificity and sensitivity of the RMOD method for the quantification of the crystallized portion of heme allowed us to describe the average hemozoin production characteristics of synchronized *P. falciparum* 3D7 parasite cultures. Our experiments revealed that the hemozoin synthesis is initiated already at the early-ring stage when the parasites are approximately 6–10 h old. These findings place the onset of hemozoin crystallization earlier than the generally assumed late-ring and early-trophozoite period. However, several studies have indicated that the formation of a feeding apparatus and the endocytic processes are commenced already at the earliest stages of intraerythrocytic development^40–42^, while other studies suggest the presence of hemoglobin degradation-derived heme already in the early ring stages (1–7 h post-invasion), and peripherally located acidified vesicular compartments containing microcrystals of hemozoin in mid-rings (12–24 h post-invasion), respectively^26,43^. According to our bulk quantitative measurements, approximately 20–25% of the total hemozoin production is completed by 20 h post-invasion—in agreement with the findings of the magnetophoretic studies of Moore *et al*.^44^—when the metabolic processes accelerate, and the cultures produce the remaining 80% of the crystals by reaching the late-trophozoite (~ 38 h post-invasion) phase. Subsequently the crystallization process appears to stagnate until the new generation of merozoites enter the mid-ring stage again. Similar kinetics for the decrease of the total hemoglobin content of the infected red blood cells have been reported by Hanssen *et al*.^45^, who found a slow and a sharper decrease during the ring and trophozoite stages, respectively, and observed the digestion process to be completed by the early-schizont stage.

Using this sensitive quantification of hemozoin production we monitored the inhibition of parasite growth by three different antimalarials with high temporal resolution. The analysis of a complete intraerythrocytic cycle under chloroquine and piperaquine pressure allowed us to obtain IC50 values for these antimalarials after various incubation times. When comparing the IC50 values calculated at intervals spanning the entire intraerythrocytic life cycle, no significant time-dependence was detected for either of them, and most importantly, reliable IC50 values were achieved already after 6-to-10 h of incubation. We observed that in some instances the exposure to low chloroquine concentrations (≤ 13 nM) slightly enhanced hemozoin production in exposed cultures compared to the drug-free controls. In contrast to the two aminoquinoline compounds, the RMOD assay revealed an increase of IC50 values of dihydroartemisinin over the course of a single erythrocytic cycle. We think this increase originates from the higher sensitivity of the very early erythrocytic stages of *Plasmodium falciparum* to artemisinins^26^. In summary, the IC50 values for the three studied antimalarial drugs were determined at several time points within the intraerythrocytic cycle by the RMOD assay.

Remarkably, the assay can work with short (6–10 h) incubation and the IC50 values are in agreement with results available in the literature from standard assays usually performed with considerably longer incubation times. Thus, it addresses the challenge to reduce the time and complexity required for assessment of drug inhibitory action^13,46–48^. Moreover, the RMOD assay proved to be applicable on cultures with parasitemia levels down to 0.1%.

In this work our primary aim was to demonstrate the applicability of the RMOD assay to test fast drug effects, often being the bottleneck for antimalarial assays. Though not directly addressed here, we see no fundamental limitations that would hinder the study of inhibition effects for long-acting drugs, such as atovaquone and pyrimethamine, by RMOD. In case of drugs with an inherently longer mechanism of action, the speed of the analysis is likely dominated by the speed of antimalarial action, and not by the response time of the method itself. Nevertheless, future studies are required to directly assess the performance of RMOD assay for drugs of the aforementioned category as well as to demonstrate the capability of RMOD for stage-specific drug inhibition, which is an important prediction of the current work.

In the current work, we demonstrate that RMOD, which does not require expensive reagents or highly trained personnel, can provide sub-cyclic temporal information about the effect of widely-used antimalarials. The high temporal resolution together with short incubation imply its potential in testing the inhibitory effect of novel drug-candidates even in a stage-specific fashion. However, to confirm the highly attractive capability of the method to stage-specific drug assessment requires further studies. While the current prototype does not allow the collective screening of a large set of samples, the measurement time for a single sample is ~ 30 s, which still allows an altogether fast analysis. On this basis, we conclude that RMOD offers a fast, reliable and practical platform for the assessment of antimalarial effect, possessing advantages that are beneficial and well-exploitable both in laboratory research and potentially in field-based settings.

## Materials and methods

### Parasite cultures and maintenance

*Plasmodium falciparum* chloroquine resistant (W2) and sensitive (3D7) strains were grown in A + erythrocytes (Hungarian National Blood Transfusion Service, Budapest, Hungary) not older than a month in complete malaria culture medium (according to the recommendations of the Malaria Research and Reference Reagent Resource Center (MR4, Bei Resources))^49^. Cultures were maintained at 5% hematocrit, 37 °C in an atmosphere of 5% O_2_, 5% CO_2_ and 90% N_2_. Cultures were synchronized regularly by applying the sorbitol and density gradient centrifugation methods^50^. Briefly, for the sorbitol method, cultures of at least 2% parasitemia and mainly ring stage parasites were incubated in 5% sorbitol (Sigma-Aldrich, Merck Group, Darmstadt, Germany) solution for 10 min at 37 °C. After washing, parasites were cultured as described above. For the density gradient centrifugation, cultures containing mostly schizont stage parasites were centrifuged with 70% Percoll (Sigma-Aldrich, Merck Group, Darmstadt, Germany) in order to separate the schizont stage parasites. The aforementioned techniques were used in combination throughout several cycles in order to achieve well-synchronized cultures. By this approach approximately ≥ 80% of the parasites fell within an 8 ± 4 h age-window at the initiation of the assays as determined by light microscopic analysis.

### Drug solutions

Chloroquine was purchased from Sigma-Aldrich. Dihydroartemisinin and piperaquine were kindly provided by Mangalam Drugs and Organics Ltd., Mumbai, India. Stock solutions of chloroquine and piperaquine were prepared in distilled water, dihydroartemisinin in pure methanol (Merck, Darmstadt, Germany). Drug concentration ranges were chosen based on the average IC50 values available in the literature^10,29–33,35,51–54^. Doubling concentrations ranging from 6 to 100 nM for chloroquine, and 6 to 486 nM for piperaquine, 0.12 to 32 nM for dihydroartemisinin were tested.

### Sample preparation for RMOD measurements

Ring-stage cultures were synchronized and washed three times in RPMI 1,640 (Biowest, Nuaillé, France) in order to remove hemozoin produced in previous parasite cycles. Cultures at 5% hematocrit and at parasitemia values presented in the main text, were incubated with antimalarial drugs or with complete medium (used for the drug-free and uninfected controls) in 96 well-plates at conditions described previously. Uninfected erythrocytes were used as controls. Samples were prepared in triplicates, i.e., three separate culture aliquots of 160 µl were prepared in separate wells for each concentration and evaluated independently. Samples were lysed by the addition of 320 μl lysis solution (13 mM NaOH and 0,5% Triton X-100; both from VWR International, Radnor, PA, USA) in distilled water, and mixed well before analysis by the RMOD method. Sampling was carried out after 6, 8, 10, 14, 18, 24, 32, 40 and 48 h of incubation during the 1% parasitemia chloroquine and dihydroartemisinin assays. For the 0.5% chloroquine assay, the following sampling point were applied after assay initiation: 8, 10, 12, 14, 16, 18, 20, 24, 32, 40 and 48 h. In the case of the 0.1% and 1% parasitemia piperaquine assays, sampling was carried out after 6, 8 and 10 h of incubation, and finally in case of the 1% parasitemia W2 strain treated with chloroquine, sampling took place 24 and 48 h after assay initiation.

The scheme of the RMOD setup, as well as the underlying physical principles of the detection, are described in detail in our former studies^15–17^. Reproduction of this experimental scheme is possible by following the described details. Briefly, the detection of hemozoin is based on its paramagnetic and dichroic properties. A strong, rotating magnetic field is used to induce the collective spinning of the crystals in the liquid medium, which results in periodic changes in the transmitted intensity of a polarized laser light. The amplitude of the intensity oscillations divided by the average transmission is defined as the magneto-optical (MO) signal measured in units of mV/V. As demonstrated in our previous studies, the MO signal is linearly proportional to the hemozoin concentration of the suspensions^14–16^.

For establishing the average hemozoin production curve of the *P. falciparum* 3D7 strain presented in Fig. 2., the MO values measured on the drug-free triplicates of the 1% parasitemia drug assays presented in the main text, together with six additional drug-free maturation assays (not presented individually) were utilized. The mean MO values displayed in Fig. 2. are the averages of 4 or 5 data points—depending on the number of available results corresponding to a given parasitic age—originating from nine independent assays, altogether. The mean age of the parasite populations at the various sampling points were determined as detailed in “Optical microscopy and smear evaluation” section.

### Optical microscopy and smear evaluation

Parasitemia and stage distributions were determined by light microscopic examination of thin blood smears prepared from the drug-free samples. Smears were stained with Kwik-Diff Stain Kit (Thermo Fisher Scientific, Waltham, MA, USA) according to product instructions^50^*.*

In order to assess the stage distribution of the cultures with high temporal resolution, an intraerythrocytic parasite maturation map (Supplementary Fig. S1.) was created from microscopy images representative of our assays, and the absolute ages of the parasites were determined based on morphologies available in the literature^25,55–58^. In all experiments presented in Figs. 2–5., smears were prepared at each time point from the drug-free samples. For each 0 h smear 100 representative parasites were categorized based on the aforementioned map, and weighted averages of the parasite ages were calculated. The ages of the cultures in later time points were estimated as the sum of the initial age and the length of incubation until sampling. Their average age was also determined at two later time points (typically 18 and 32 h) by the categorization of at least 30 parasites. We estimate a ± 6 h uncertainty for the assessment of the mean ages, which includes the inaccuracy of parasite categorization and the width of the stage distribution characterizing the cultures.

### Determination of IC50 values

In order to determine the IC50 values, the optical signals measured on culture aliquots dosed with different drug concentrations, after various incubation times, were fitted with a variable-slope sigmoidal dose–response formula $\left(y=A1+\frac{A2-A1}{1+{10}^{\left(\mathrm{log}\left[IC50\right]-x\right)\cdot p}}\right)$ using Origin Pro 9 software (OriginLab Corporation, Northampton, MA, USA)^59^.

### Special note

With requests for testing new antimalarial drug candidates or copies of the RMOD device for the purpose of collaborative research projects, please contact vertessy@mail.bme.hu and istvan.kezsmarki@physik.uni-augsburg.de.

## Supplementary information

Supplementary file1
