# Supplementary material for: Rapid and quantitative antimalarial drug efficacy testing via the magneto-optical detection of hemozoin
Source: Sci Rep. 2020 Aug 20;10:14025. doi: 10.1038/s41598-020-70860-y (PMC7441145; doi:10.1038/s41598-020-70860-y)
Supplement: Supplementary file 1 — Supplementary file1 [file 41598_2020_70860_MOESM1_ESM.pdf]

Supplementary materials of Rapid and quantitative antimalarial drug efficacy testing via the magneto-optical detection of hemozoin

Petra Molnár[1,2,4*], Ágnes Orbán[1,2], Richard Izrael[1,3,4], Réka Babai[4], Lívia Marton[1], Ádám Butykai[2], Stephan Karl[5,6], Beáta G. Vértessy[1,4*], István Kézsmárki[1,2,7*]

[1]Malaria Research Laboratory, Institute of Enzymology, Research Centre for Natural Sciences, 1117 Budapest, Hungary;

[2] Department of Physics, BME Budapest University of Technology and Economics, 1111 Budapest, Hungary;

[3] Doctoral School of Multidisciplinary Medical Sciences, University of Szeged, 6720 Szeged, Hungary;

[4] Department of Applied Biotechnology and Food Sciences, BME Budapest University of Technology and Economics, 1111 Budapest, Hungary;

[5] Australian Institute of Tropical Health and Medicine, James Cook University, 1/14-88 McGregor Road, Smithfield QLD 4870 Australia

[6] Vector-borne Diseases Unit, PNG Institute of Medical Research, P.O. Box 378, Madang 511, Madang Province, Papua New Guinea

[7] Experimental Physics 5, Center for Electronic Correlations and Magnetism, Institute of Physics, University of Augsburg, 86159 Augsburg, Germany;

*corresponding authors, e-mail addresses: molnar.petra@ttk.mta.hu; vertessy@mail.bme.hu; istvan.kezsmarki@physik.uni-augsburg.de

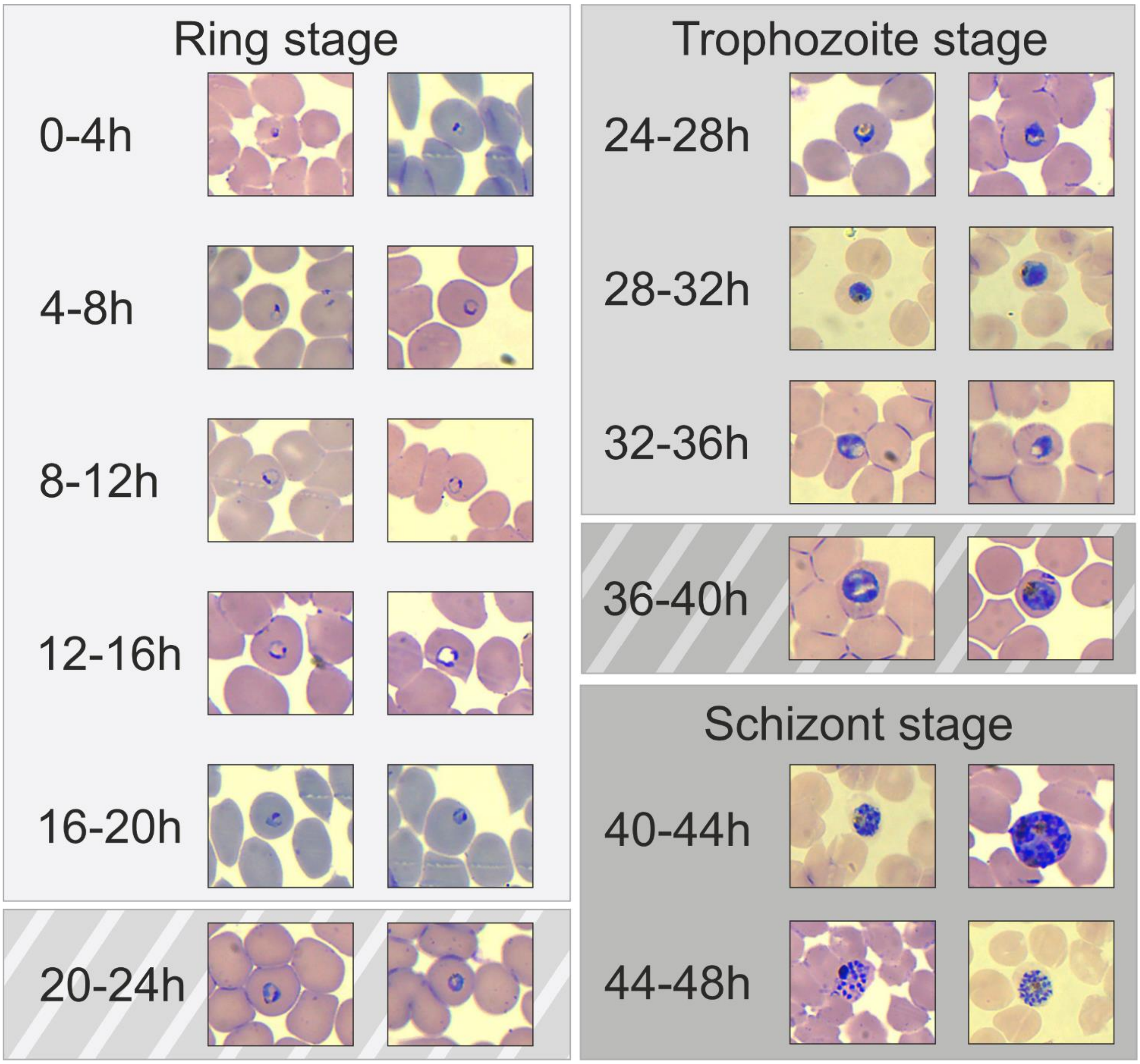

Supplementary Figure S1. - An intraerythrocytic parasite maturation map. The map follows the maturation of *P. falciparum* 3D7 parasites in laboratory cultures[1]–[5]. For details, see Materials and Methods Section.

Supplementary Table S1. – IC50 values for chloroquine (CQ), piperaquine (PPQ) and dihydroartemisinin (DHA) obtained by different methods. Parasitemia values of the assays are also included, where applicable.

		RMOD	[$_3$H]-Hypoxanthine incorporation	SYBR Green	HRP2 ELISA	pLDH DELI
**IC50**	**CQ:**	0.5%:29.15 ±2.71 nM<br>1%: 34.68 ±5.28 nM	0.5%: 6,5 nM [6]<br>0.5%: 15,7 nM [7]<br>0.5%: 29,6 nM [8]<br>0.5%: 9,73 nM [9]	0.5%: 8,1 nM [10]<br>1%: 15,11 nM [11]<br>0.5%: 16,8 nM [12]<br>0.5%: 11,54 nM [13]	0.5%: 7,5 nM [10]<br>0.5%: 21,91 nM [12]<br>0.5%: 9,68 nM [13]<br>0.5%: 22 nM [14]<br>1%: 18 nM [15]	0,5%: 15,0 nM [13]<br>1%: 15 nM [15]<br>0,5%: 40,5 nM [16]<br>15,7 nM [17]
	**PPQ:**	0.1%: 30.61±4.01 nM<br>1%: 72±1.59 nM	0.5%: 27 nM [6]<br>0.5%:16,9 nM [8]<br>1%: 36,9 nM [18]	1%: 14,88 nM [11]<br>0.5%: 18,5 nM [12]<br>46,3 nM [19]	0.5%: 21,0 nM [12]<br>1%: 29 nM [15]	1%: 30 nM [15]<br>0,1%: 15,1 nM [20]
	**DHA:**	1%: 2.13±0.87 nM	0.5%: 2,0 nM [6]<br>0.5%: 5,27 nM [7]<br>0.5%: 4,2 nM [8]	1%: 2,11 nM [11]<br>0.5%: 2,97 nM [12]	0.5%: 6,00 nM [12]<br>1%: 6 nM [15]	1%: 6,3 nM [15]<br>0,1%: 2,6 nM [20]

Supplementary References

1. Kozicki, M. *et al.* The ring-stage of Plasmodium falciparum observed in RBCs of hospitalized malaria patients. *Analyst* **140**, 8007–8016 (2015).

2. Moura, P. A., Dame, J. B. & Fidock, D. A. Role of Plasmodium falciparum digestive vacuole plasmepsins in the specificity and antimalarial mode of action of cysteine and aspartic protease inhibitors. *Antimicrob. Agents Chemother.* **53**, 4968–4978 (2009).

3. Grüring, C. *et al.* Development and host cell modifications of Plasmodium falciparum blood stages in four dimensions. (2011) doi:10.1038/ncomms1169.

4. Silamut, K. *et al.* A quantitative analysis of the microvascular sequestration of malaria parasites in the human brain. *Am. J. Pathol.* **155**, 395–410 (1999).

5. White, N. J. Qinghaosu (artemisinin): The price of success. *Science* vol. 320 330–334 (2008).

6. Mwai, L. *et al.* In vitro activities of piperaquine, lumefantrine, and dihydroartemisinin in Kenyan plasmodium falciparum isolates and polymorphisms in pfcrt and pfmdr1. *Antimicrob. Agents Chemother.* **53**, 5069–5073 (2009).

7. Duraisingh, M. T., Roper, C., Walliker, D. & Warhurst, D. C. Increased sensitivity to the antimalarials mefloquine and artemisinin is conferred by mutations in the pfmdr1 gene of Plasmodium falciparum. *Mol. Microbiol.* **36**, 955–961 (2000).

8. Wong, R. P. M. *et al.* Desbutyl-lumefantrine is a metabolite of lumefantrine with potent in vitro antimalarial activity that may influence artemether-lumefantrine treatment outcome. *Antimicrob. Agents Chemother.* **55**, 1194–1198 (2011).

9. Vivas, L. *et al.* Antimalarial efficacy and drug interactions of the novel semi-synthetic endoperoxide artemisone in vitro and in vivo. *J. Antimicrob. Chemother.* **59**, 658–665

(2007).

10. Bacon, D. J. *et al.* Comparison of a SYBR green I-based assay with a histidine-rich protein II enzyme-linked immunosorbent assay for in vitro antimalarial drug efficacy testing and application to clinical isolates. *Antimicrob. Agents Chemother.* **51**, 1172–1178 (2007).

11. Pooja, A., A. R., A., C. R., P. & Kumkum, S. In vitro susceptibility of Indian Plasmodium falciparum isolates to different antimalarial drugs & antibiotics. *Indian Journal of Medical Research* 622–628 (2017) doi:10.4103/ijmr.IJMR_1688_15.

12. Chaorattanakawee, S. *et al.* Direct comparison of the histidine-rich protein-2 enzyme-linked immunosorbent assay (HRP-2 ELISA) and malaria SYBR green i fluorescence (MSF) drug sensitivity tests in Plasmodium falciparum reference clones and fresh ex vivo field isolates from Cambodia. *Malar. J.* **12**, 1–11 (2013).

13. Wein, S. *et al.* Reliability of antimalarial sensitivity tests depends on drug mechanisms of action. *J. Clin. Microbiol.* **48**, 1651–1660 (2010).

14. Rebelo, M. *et al.* A Novel Flow Cytometric Hemozoin Detection Assay for Real-Time Sensitivity Testing of Plasmodium falciparum. *PLoS One* **8**, (2013).

15. Chaorattanakawee, S. *et al.* Measuring ex vivo drug susceptibility in Plasmodium vivax isolates from Cambodia. *Malar. J.* **16**, 1–13 (2017).

16. Kaddouri, H., Nakache, S., Houzé, S., Mentré, F. & Le Bras, J. Assessment of the drug susceptibility of Plasmodium falciparum clinical isolates from Africa by using a Plasmodium lactate dehydrogenase immunodetection assay and an inhibitory maximum effect model for precise measurement of the 50-percent inhibitory conc. *Antimicrob. Agents Chemother.* **50**, 3343–3349 (2006).

17. Druilhe, P., Moreno, A., Blanc, C., Brasseur, P. H. & Jacquier, P. *A colorimetric in vitro drug sensitivity assay for Plasmodium falciparum based on a highly sensitive double-site lactate dehydrogenase antigen-capture enzyme-linked immunosorbent assay*. *Am. J. Trop. Med. Hyg* vol. 64 (2001).

18. Fivelman, Q. L., Adagu, I. S. & Warhurst, D. C. Effects of Piperaquine, Chloroquine, and Amodiaquine on Drug Uptake and of These in Combination with Dihydroartemisinin against Drug-Sensitive and-Resistant Plasmodium falciparum Strains. *Antimicrob. Agents Chemother.* **51**, 2265–2267 (2007).

19. Murithi, J. M. *et al.* Combining Stage Specificity and Metabolomic Profiling to Advance Antimalarial Drug Discovery. *Cell Chem. Biol.* **27**, 158-171.e3 (2020).

20. Woodrow, C. J. *et al.* Comparison between flow cytometry, microscopy, and lactate dehydrogenase-based enzyme-linked immunosorbent assay for plasmodium falciparum drug susceptibility testing under field conditions. *J. Clin. Microbiol.* **53**, 3296–3303 (2015).
